# Biocatalytic quantification of α‐glucan in marine particulate organic matter

**DOI:** 10.1002/mbo3.1289

**Published:** 2022-05-26

**Authors:** Nicola Steinke, Silvia Vidal‐Melgosa, Mikkel Schultz‐Johansen, Jan‐Hendrik Hehemann

**Affiliations:** ^1^ MARUM—Center for Marine Environmental Sciences, Faculty of Biology and Chemistry University of Bremen Bremen Germany; ^2^ Max Planck Institute for Marine Microbiology Bremen Germany

**Keywords:** algae, enzymatic hydrolysis, glucans, marine particulate organic matter, polysaccharides, quantification

## Abstract

Marine algae drive the marine carbon cycle, converting carbon dioxide into organic material. A major component of this produced biomass is a variety of glycans. Marine α‐glucans include a range of storage glycans from red and green algae, bacteria, fungi, and animals. Although these compounds are likely to account for a high amount of the carbon stored in the oceans they have not been quantified in marine samples so far. Here we present a method to extract and quantify α‐glucans (and compare it with the β‐glucan laminarin) in particulate organic matter from algal cultures and environmental samples using sequential physicochemical extraction and enzymes as α‐glucan‐specific probes. This enzymatic assay is more specific and less susceptible to side reactions than chemical hydrolysis. Using HPAEC‐PAD to detect the hydrolysis products allows for a glycan quantification in particulate marine samples down to a concentration of ≈2 µg/L. We measured glucans in three cultured microalgae as well as in marine particulate organic matter from the North Sea and western North Atlantic Ocean. While the β‐glucan laminarin from diatoms and brown algae is an essential component of marine carbon turnover, our results further indicate the significant contribution of starch‐like α‐glucans to marine particulate organic matter. Henceforth, the combination of glycan‐linkage‐specific enzymes and chromatographic hydrolysis product detection can provide a powerful tool in the exploration of marine glycans and their role in the global carbon cycle.

## Graphical abstract

α‐Glucans can be quantified alongside laminarin in marine particulate organic matter samples using structure‐specific hydrolytic enzymes in combination with glucose detection by high‐performance anion‐exchange chromatography and pulsed amperometric detection. This enzymatic method is a new tool for the characterization and quantification of specific algal glycans in the ocean, which is important to understanding microbial carbon cycling and carbon sequestration in the marine environment.

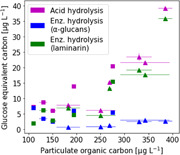

## 1 INTRODUCTION

Marine microalgae and other autotrophic organisms in the oceans are estimated to contribute about half of the global primary production (Field et al., 1998). Around 50% of the carbon that is fixed by marine autotrophs is directly consumed and eventually reconverted into carbon dioxide by bacteria (Buchan et al., 2014). A fraction of the biomass escapes microbial degradation and accumulates over time, becoming part of sinking particles that are exported to the ocean floor (Hedges et al., 2001; Vidal‐Melgosa et al., 2021, 2022). Glycans constitute the majority of organic matter produced by algae (Myklestad, 1974), and are therefore a central part of the complex marine food web. Characterization and quantification of algal glycans represent an important task to understand microbial carbon cycling and sequestration in the marine environment (Engel et al., 2004; Hedges et al., 2001).

Glycans are complex and often nonlinear polymers built from numerous possible monosaccharides and chemical modifications (Laine, 1994). This complexity makes the structural analysis of glycans challenging and the analysis of glycans in marine dissolved or particulate organic matter even more difficult as the glycans are present in complex mixtures and at often low individual concentrations. The lack of structure‐specific analytical tools for marine glycan samples leads to these glycans being commonly identified and quantified by their monosaccharide content after chemical lysis (Engel & Händel, 2011; Panagiotopoulos & Sempéré, 2005). This nonselective acid hydrolysis destroys information about glycosidic linkages and some chemical modifications. It is therefore not suitable to unequivocally identify different types of glycans. While there are derivatization methods in combination with mass spectrometry to distinguish sugar linkages (Galermo et al., 2018; Price, 2007), there is still a fundamental lack of knowledge about the structures and functions of marine polysaccharides and the marine carbon cycle (Hedges et al., 2001).

Glucans are glycans derived from d‐glucose residues. Some glucans like starch, glycogen, and laminarin are energy storage glycans while other glucans like cellulose are structural polymers (Painter, 1983; Suzuki & Suzuki, 2013). Acid hydrolysis makes these glycans of different biological origins indistinguishable and obscures insight into their roles in the marine carbon cycle.

α‐Glucans include a range of different types of polysaccharides, most of them containing α‐1,4 and α‐1,6‐glycosidic linkages. Starch is a mixture of the linear α‐1,4‐linked amylose and the branched amylopectin composed of α‐1,4‐ and α‐1,6‐glycosidic bonds (Imberty et al., 1991). Starch is commonly known as a storage glycan for terrestrial plants but it is also found in green algae. Floridean starch is a storage α‐glucan in red algae similar to amylopectin but with a higher degree of α‐1,6‐branching (Yu et al., 2002). Glycogen is another highly α‐1,6‐branched storage α‐glucan found in animals, fungi, and bacteria (Ball & Morell, 2003). Although the amount of α‐glucans in the ocean is unknown, analysis of marine bacterial polysaccharide degradation pathways (Fang et al., 2019; Kappelmann et al., 2019) indicates that α‐glucans are an important carbohydrate source for marine bacteria.

Glycan‐specific glycoside hydrolases (GHs) can be applied to identify and quantify α‐glucans in the ocean. There are several well‐characterized starch‐specific glycoside hydrolases (α‐amylases and amyloglucosidases) that have been used to quantify starch in food (McCleary et al., 1994, 2002), yet a more sensitive method for starch quantification in marine environmental samples is missing.

In this study, a set of commercial enzymes was adapted into an assay for the quantification of α‐glucans in different types of microalgae and unprocessed marine environmental samples. This assay was tested in parallel with a previously developed laminarin assay (Becker et al., 2017) on different types of α‐glucans and other polysaccharides to explore possible side reactions and compare the enzymatic hydrolysis (EH) with the commonly used acid hydrolysis (AH). Additionally, the detection range of the assay was tested using two types of glucose detection, the spectrophotometric PAHBAH assay, and HPAEC‐PAD. Furthermore, the extraction of glucans from different types of microalgae was optimized. The α‐glucan and laminarin content of these microalgae was tested on different days of algal growth and compared to the total particulate organic carbon (POC) in these samples. Finally, the assay was used on two sets of particulate organic matter (POM) samples from the North Sea (spring 2020) and the western North Atlantic Ocean (spring 2019) to demonstrate that this method allows for a quantification of low concentrations of α‐glucans alongside laminarin in crude marine samples.

## 2 MATERIALS AND PROCEDURES

### 2.1 Algal cultures

To test different extraction protocols and enzymatic hydrolysis of algal glucans, three types of microalgae were used as model organisms for green and red algae and diatoms, respectively: The green microalga *Ostreococcus tauri* (Ral et al., 2004) was grown in L1 medium (Guillard, 1983; Guillard & Hargraves, 1993), and the red microalgae *Porphyridium purpureum* and the diatom *Thalassiosira weissflogii* were grown in NEPCC medium (Harrison et al., 1980).

Algae cultures were kept at a constant temperature of 15°C, with a 12‐h/12‐h light/dark cycle, without stirring and irradiated with ≈140 μmol photons $m^{-2}s^{-1}$.

For glucan extraction tests, duplicate T75 flasks with 400 ml growth medium were inoculated with 5 ml of 7‐days old algal cultures. After 20 days the material was filtered at 200 mbar on a combusted (450°C, 4 h) 25 mm GF/F glass microfiber filter (using 20 ml of algal culture per filter). The filters were stored at −20°C until extraction.

For the α‐ and β‐glucan quantifications algae were cultivated in triplicate batch cultures. A total volume of 250 ml in T75 suspension cell culture flasks was inoculated with 5 ml of a 25‐ml culture that had been grown for 7 days. Ten to twenty‐five milliliters of each culture were taken over 20 days and filtered as described above.

### 2.2 Environmental sample collection

Sampling in the North Atlantic Ocean (40°53.7′ N, 60°11.9′ W) was carried out in May 2019 and in the North Sea (54°11.3′ N, 7°54.0′ E) in April 2020. In both locations, surface seawater was collected and directly filtered through precombusted (400°C, 4 h) GF/F glass filters with 142 mm diameter (Whatman glass microfiber filters, WHA1825142; Sigma‐Aldrich). For the Atlantic Ocean samples, 50 L seawater was filtered through each GF/F filter with a peristaltic pump (Watson Marlow 630 S) at 40 rpm. Filters were stored at −80°.C until further analysis. For the North Sea samples, 15 L seawater was filtered through each GF/F filter with an air pressure pump (Flojet G57; ITT Industries) at ≈0.2–0.5 bar. Filters were stored at −30°C until further analysis.

### 2.3 Extraction of glucans from particulate algae material

The filters were cut into equally sized pieces and subjected to extraction tests. One filter piece was kept as a nonextracted reference. Each extraction was tested using filter‐part triplicates of three different filters. Afterward, the extracted and non‐extracted filter parts were put under AH conditions and the glucose content of the acid extract was tested using HPAEC‐PAD. The extraction conditions tested include water or 1M NaOH extractions at 99°C or in a sonication bath, different extraction times, and sequential extractions.

### 2.4 Acid hydrolysis

Glycan samples in solution or algal samples on glass fiber filters were incubated in 1 M HCl for 24 h at 100°C in sealed glass ampoules (Engel & Händel, 2011) to chemically hydrolyze glycans. Afterward, 100 μl of each acid solution was evaporated using a speed‐vac (RVC 2‐18 CDplus HCl resistant; Christ) and resuspended in 100 μl buffer or Milli‐Q water.

### 2.5 Enzymatic hydrolysis

α‐Glucans were hydrolyzed using α‐amylase (*Aspergillus oryzae*) obtained from Megazyme and amyloglucosidase (*Aspergillus niger*) from Merck. The stock concentration was 1 U/μl ($=1\;\mu \;\text{mol}\;\min^{-1}\;{\mathrm{\mu l}}^{-1}$) for both enzymes.

One unit of α‐amylase is the amount of enzyme required to release 1 μmole of *p*‐nitrophenol from blocked *p*‐nitrophenyl‐maltoheptaoside per minute (in the presence of excess α‐glucosidase), pH 5.4 at 40°C (Megazyme, 2022). One unit of amyloglucosidase activity is defined as 1.0 mg of glucose hydrolyzed from starch in 3 min at pH 4.5 at 55°C (Merck, 2022).

Samples containing polysaccharide standards or extracted glucans were split into six subsamples: three each for enzyme‐hydrolyzed and nonhydrolyzed controls.

Triplicate subsamples of 90 μl aqueous glycan extract were mixed with 10 μl sodium acetate buffer (1 M, pH 4.5), 0.4 μl α‐amylase (in 3.2 M ammonium sulfate), 0.9 μl amyloglucosidase (0.1 M sodium acetate), and 1 μl BSA solution (100 mg/ml).

Triplicate nonhydrolyzed subsamples were prepared in the same way but contained 0.4 μl 3.2 M ammonium sulfate and 0.9 μl 0.1 M sodium acetate instead of enzymes. Reaction contents were mixed and incubated for 35 min at 50°C in a heat block. Then, the enzyme reactions were inactivated for 5 min at 99°C, centrifuged (10,000 rpm for 30 s), and cooled on ice.

Subsamples used for laminarin quantification (90 μl) were hydrolyzed using 90 μl aqueous glycan solution, 10 μl 500 mM MOPS buffer (pH 7.0), and laminarinases as previously described (Becker et al., 2017).

### 2.6 PAHBAH reducing sugar assay

Photometric quantification of polysaccharide hydrolysis products was performed using the PAHBAH reducing sugar assay (Lever, 1972). One milliliter of a freshly prepared 9:1 (v/v) mixture of PAHBAH reagent A (0.3 M 4‐hydroxybenzhydrazide, 0.6 M HCl) and PAHBAH reagent B (48 mM trisodium citrate, 10 mM ${\text{CaCl}}_{2}$, 0.5 M NaOH) was added to 0.1 ml of polysaccharide standard or extract sample. Hydrolyzed and non‐hydrolyzed EH samples were used without further cleaning. After incubation for 5 min at 99°C, the samples were cooled on ice and the absorbance at 410 nm was measured using 10 mm pathlength semi‐micro cuvets and a BioSpectrometer (Eppendorf). PAHBAH reaction mix A/B (9:1, v/v) was used as a blank.

**Figure A1:**
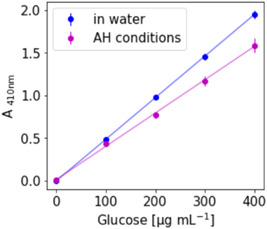
PAHBAH signal of glucose: Different concentrations of glucose either directly dissolved in water or subjected to AH conditions. AH, acid hydrolysis.

Glucose was used as a quantification standard. The glucose standards used for quantification were treated the same way as the respective samples. This corrects for absorbance changes in the assay caused by the addition of specific buffers and enzymes, or by AH (Figure A1).

### 2.7 HPAEC‐PAD

All samples for HPAEC‐PAD (high‐performance anion‐exchange chromatography with pulsed amperometric detection) were filtered using a 0.2 μm Spin‐X filter and transferred into an HPLC vial with a micro‐insert. The monosaccharide (and specifically glucose) content was determined using HPAEC‐PAD with a Dionex CarboPac PA10 column (Thermo Scientific) and monosaccharide mixes as standards for calibration. The oligosaccharide content was determined using Dionex CarboPac PA200 and PA100 columns (Thermo Scientific) and appropriate malto‐ and laminarin oligosaccharides (Megazyme).

### 2.8 Glucan quantification

Glucans in extracted samples were quantified as glucose equivalents based on a calibration curve generated with glucose standards.

The glucose signals were inferred from PAHBAH absorbance readings or integrated peak areas from HPAEC‐PAD chromatograms. Each hydrolyzed sample or non‐hydrolyzed control was measured in triplicates. To correct for background signals in the enzymatically digested samples, the glucose values detected for non‐hydrolyzed samples were subtracted. To measure the total glucose or reducing sugar concentration after AH no background correction with non‐hydrolyzed samples was applied.

The glucan concentrations in filter extracts were normalized by extraction volume, filter size, and filtration volume.

### 2.9 Particulate organic carbon quantification

Particulate organic matter from cultured algae or environmental samples were both filtered on glass fiber filters. Defined pieces of these filters were punched out in triplicates and subjected to an acidic atmosphere with concentrated HCl for 24 h in a desiccator to remove inorganic carbon. Afterward, the filters were dried for 24 h at 60°C and wrapped in combusted tin foil. The carbon quantification was performed by an elemental analyzer (vario MICRO cube; Elementar Analysensysteme) using sulfanilamide as a calibration standard.

## 3 ASSESSMENT

### 3.1 Optimal conditions for enzymatic α‐glucan hydrolysis

**Figure A2:**
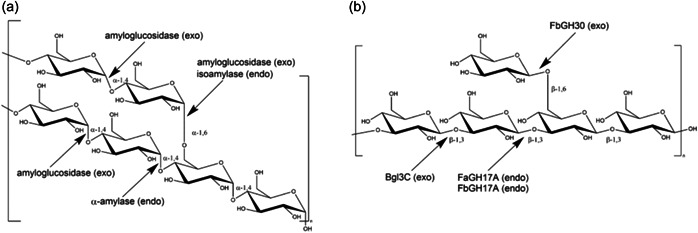
Partial structures of α‐ and β‐glucans and sites of glycoside hydrolases activities: (a) A branched α‐glucan structure as in amylopectin or glycogen with an α‐1,4‐linked d‐glucose linear chain and α‐1,6 branches. The GH13 endo‐enzymes α‐amylase and isoamylase hydrolyze α‐1,4 linkages and branching α‐1,6 linkages respectively. The exo‐acting enzyme amyloglucosidase (GH15) is active on terminal α‐1,4 or α‐1,6 glycosidic bonds. (b) The β‐glucan laminarin consists of a β‐1,3‐d‐glucose polysaccharide main chain with β‐1,6 linked single residue d‐glucose side chains. FbGH30 is an exo‐acting β‐1,6 glucanase of the GH30 family hydrolyzing the branching glucose monomers attached to the laminarin main chain. FaGH17A and FbGH17A are two endo‐acting β‐1,3‐glucanases of the GH family 17, which act within the β‐1,3‐linked laminarin backbone (Becker et al., 2017; Unfried et al., 2018). Bgl3C further hydrolyzes the β‐1,3‐d‐linked oligos into glucose in an exo‐acting manner (Nelson et al., 2017).

**Figure A3:**
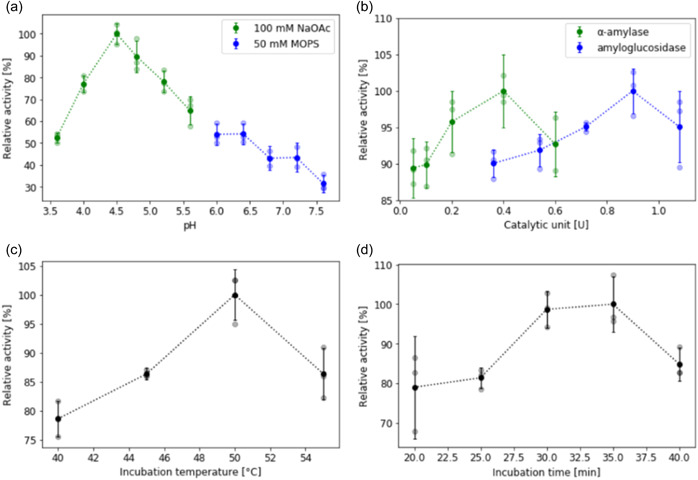
Optimal conditions for enzymatic α‐glucan hydrolysis: enzymatic activity rates were measured using the PAHBAH reducing sugar assay with 100 μg/ml100μ starch. The greatest activity observed was set as the 100% reference value for each parameter tested. (a) Comparison of different buffer and pH conditions. (b) Comparison of different enzyme concentrations per sample. (c) Comparison of different incubation temperatures. (d) Comparison of different incubation times.

**Figure A4:**
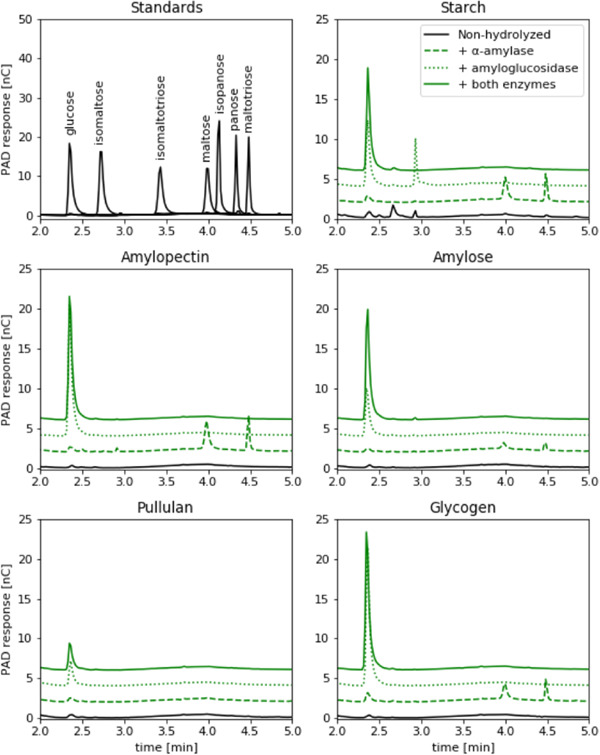
Enzymatic hydrolysis assays for α‐glucans produce glucose: HPAEC‐PAD chromatograms (PA200 column) of (top left) maltose standards and glucose, α‐glucans with and without enzymatic treatment: (top left) starch, (middle left) amylopectin, (middle right) amylose, (bottom left) pullulan and (bottom right) glycogen. Chromatograms of enzymatically hydrolyzed α‐glucans were moved by 2, 4, and 6 to improve clarity.

Based on an assay for amylose and amylopectin quantification in cereal starches and flours (Megazyme, 2018), a similar assay for total α‐glucan quantification (without amylose/amylopectin separation) was developed and optimized for a sample volume of 100 μl, a starch concentration of 100 μg/ml (from corn; Sigma‐Aldrich) and hydrolysis product detection using the PAHBAH reducing sugar assay (Lever, 1972). Optimal EH was achieved by incubating a 100 μl starch sample in 100 mM sodium acetate buffer (pH 4.5) for 35 min at 50°C in the presence of 0.4 U of the *endo*‐acting α‐amylase (from *Aspergillus oryzae*, Megazyme) and 0.9 U of the *exo*‐acting amyloglucosidase (from *A. niger*; Merck). Under these conditions, PAHBAH reducing sugar signals were maximal (Figures A2, A3). Subsequently, the complete hydrolysis of different α‐glucan polysaccharides, namely starch, glycogen, amylose, or amylopectin down to glucose monosaccharides was confirmed by HPAEC‐PAD (Figure A4).

### 3.2 Enzymatic hydrolysis of α‐glucans is more effective than acid hydrolysis

**Figure 1:**
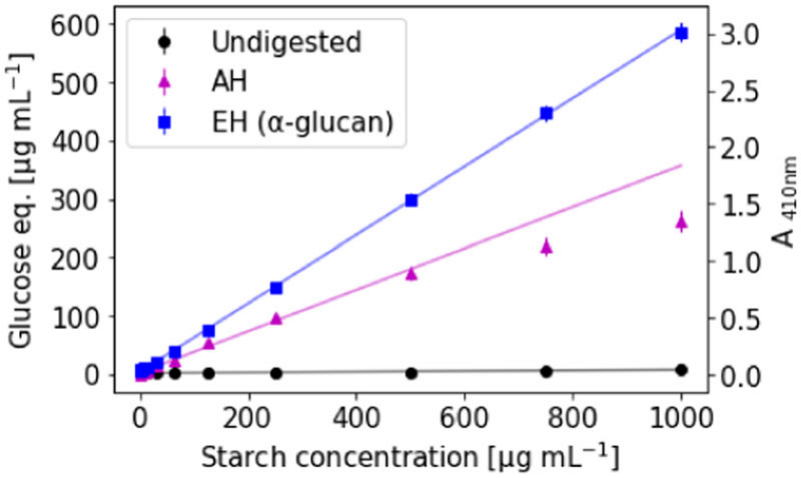
Enzymatic hydrolysis is more effective than acid hydrolysis for starch: Enzymatic hydrolysis (EH) products of starch, acid hydrolysis (AH) products of starch, nondigested starch, and glucose as calibration standards were measured in α‐glucan assay buffer (100 mM sodium acetate buffer, pH 4.5) using the PAHBAH reducing sugar assay. $A_{410\text{nm}}$ signals above 1.9 were measured in diluted samples. Error bars denote standard deviation (*n* = 3, technical replicates). Solid lines represent regression lines. A plot zoomed‐in on starch concentrations <100 μg/ml can be found in the appendix (Figure A5).

The enzymatic α‐glucan hydrolysis was tested on different starch concentrations and compared to AH using the PAHBAH reducing sugar assay as a glucose detection method (Figure 1). The limit of quantification was found to be ≈11 μg/ml starch, both for AH, and for EH in combination with the PAHBAH assay. However, it was found that the EH of starch (1–1000 μg/ml) generated a higher photometric signal than AH suggesting that more complete polysaccharide hydrolysis was achieved or that EH is less prone to generate side products (Cai et al., 2014). Especially at higher starch concentrations above 250 μg/ml the AH glucose signal visibly flattened, resulting in a nonlinear regression line. In contrast, EH of starch followed a linear regression model for all measured starch concentrations up to 1 mg/ml.

Complete hydrolysis of 1 g of a glucan into glucose would theoretically yield approximately 1.1 g of glucose by the addition of 1 water molecule per hydrolyzed glucose molecule. In comparison, Figure 1 shows that EH is not complete, as 1 mg/ml of starch yields 0.6 mg/ml of glucose equivalents. This result is probably due to the water insolubility of some starch particles and incomplete enzymatic hydrolysis.

It should be noted that for these measurements, the glucose calibration curve was prepared in water. Under AH conditions the glucose signals in the calibration curve were (≈−20%) lower (Figure A1). These data indicate that lower glucose signals in acid hydrolyzed starch samples could partially be due to acid‐catalyzed conversion of glucose. But this loss does not account for the total difference in the signal of acid and enzymatic starch hydrolysis and our results also indicate that AH of starch is less complete than EH.

Overall, these results show that AH—the traditional quantification method—may underestimate the true glycan content in environmental samples due to incomplete glycan hydrolysis and possible side reactions reducing the detectable monosaccharide content.

### 3.3 Enzymatic hydrolysis of α‐glucans is specific

**Figure 2:**
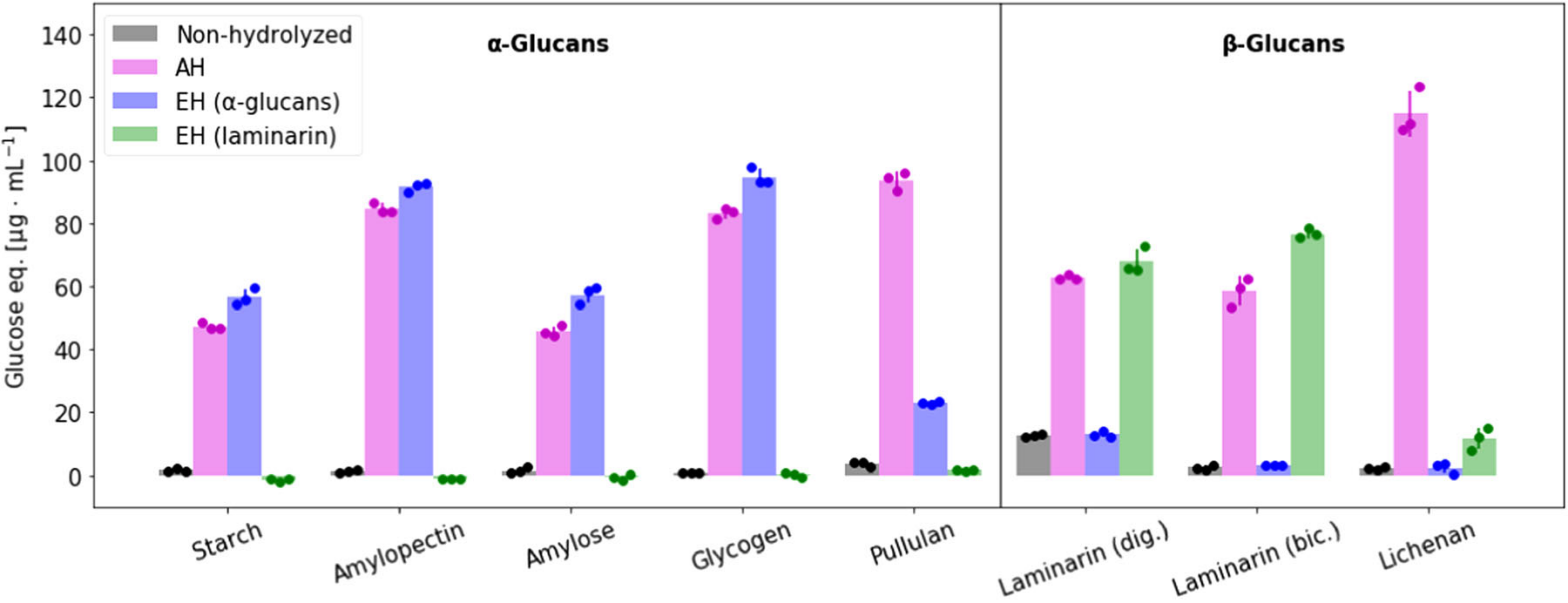
Enzymatic hydrolysis of α‐glucans is specific and more effective than acid hydrolysis: Hydrolysis efficiency of different polysaccharides (100 μg/ml) using acid hydrolysis (AH) compared to enzymatic hydrolysis (EH) using the α‐glucan or laminarin assay. All tested polysaccharides are listed in Table A1. Only the results of polysaccharides that could be hydrolyzed by either of the enzymatic assays are depicted. Data points represent individual samples, and error bars denote standard deviation (*n* = 3, technical replicates).

To explore the specificity of the enzymatic α‐glucan assay compared with AH, 14 commercially available polysaccharides were tested. The enzymatic laminarin assay was included as a control. Figure 2 shows the reducing sugar assay results for the eight polysaccharides that could be hydrolyzed by either of the enzymatic assays compared to AH. Additionally tested polysaccharides that could only be hydrolyzed by acid are listed in Table A1.

The α‐glucan assay is able to hydrolyze all polysaccharides containing α‐glucan 1‐4‐ and 1‐6‐linkages, namely amylopectin, amylose, glycogen, and pullulan.

The reducing sugar signal of enzymatically hydrolyzed amylopectin and glycogen equals approximately 100 μg/ml glucose, indicating complete hydrolysis to glucose. Amylose is poorly soluble in water, and therefore, only 60 μg/ml glucose equivalents, presumably produced by shorter‐chain amylose, could be measured using EH.

Similar to the results in Figure 1 the concentration of glucose hydrolysis products is often higher for the α‐glucan enzymatic assay than for the AH with the notable exception of pullulan, which is less effectively hydrolyzed by the enzymatic assay than by AH. Pullulan is comprised of α‐1‐4‐maltotriose units linked by α‐1‐6‐linkages and should—in theory—be completely hydrolyzed by the applied *endo*‐1‐4‐α‐amylase and *exo*‐1,4/1,6‐α‐amyloglucosidase. However, as the observed reducing sugar signal is only slightly increased by the application of these enzymes, it can be assumed that this α‐amylase needs a longer uninterrupted α‐1‐4‐linked glucose chain to be more active. Thus, the α‐glucan assay can mainly be used to identify and quantify starch‐like α‐glucans with 1‐4 chains and optional 1‐6 branches.

While the laminarin assay is effective on both laminarins tested and the amount of released glucose is similar and slightly higher than for AH, the only other polysaccharide being partially hydrolyzed by this assay is lichenan. As lichenan is comprised of β‐1‐3 and β‐1‐4 linked glucose units and some of the applied laminarinases have a β‐1‐3 activity (Becker et al., 2017), this small side reaction is to be expected. However, the results show that the laminarin assay cannot detect β‐glucan from barley and any of the tested α‐glucans.

Overall, these results demonstrate that a combination of the enzymatic assays can distinguish the important storage glucans; starch‐like α‐1,4/1,6‐glucans and laminarin—something that cannot be achieved using traditional chemical hydrolysis.

### 3.4 Microalgal glucans can be extracted using hot water and sonication

The use of enzymatic assays to quantify glucans in marine POM requires that the glucans can be extracted from environmental samples. Particulate microalgal material was used to test different extraction protocols. The extraction efficiency was determined by measuring the ratio of glucose detected by HPAEC‐PAD on extracted and nonextracted filter pieces after AH conditions, assuming that these conditions are sufficient for a complete glucan extraction and hydrolysis. For the extraction of glucans from filters, we tested different combinations of water and NaOH incubations, with and without sonication.

**Figure 3:**
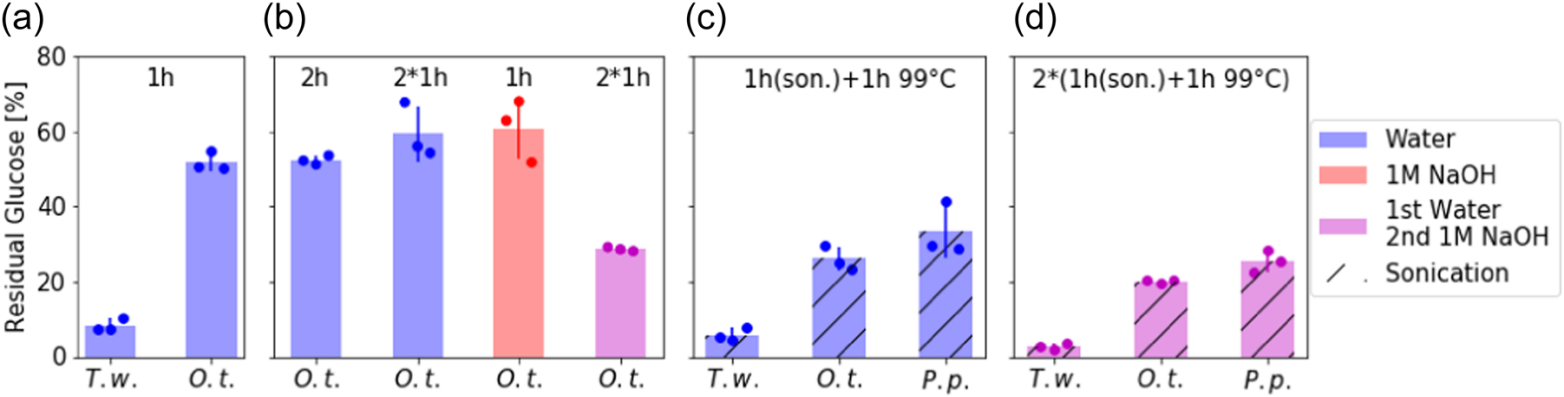
α‐Glucans can be extracted from microalgae: residual glucose content of algal POM on glass fiber filters after extractions (see below) and subsequent AH was determined using HPAEC‐PAD. (a) Hot water extraction (1 h, $99^{\circ }$C) of *Thalassiosira weissflogii* (*T. w*.) and *Ostreococcus tauri* (*O. t*.) POM. (b) *O. t*. extractions with a longer incubation time (water, 2 h, 99°C), sequential double extraction (water, 2×1 h, $99^{\circ }$C), alkaline extraction (1 M sodium hydroxide, 1h, 99°C) or sequential water (1 h, 99°C) and alkaline (1 M sodium hydroxide, 1 h, 99°C) extraction. (c) Hot water extraction of microalgae (*T. w*., *O. t*., *Porphyridium purpureum* [*P. p*.]) with prior sonication treatment (1 h sonication bath, 1 h, $99^{\circ }$C), (d) additional subsequent alkaline extraction (1 M sodium hydroxide, 1 h sonication bath, 1 h, $99^{\circ }$C). Individual data points are shown in darker colors, error bars denote standard deviation (*n* = 3, technical replicates). AH, acid hydrolysis; POM, particulate organic matter.

Figure 3 shows the results of these extraction condition tests as the proportion of (total) glucose that remains in the filters after extraction. Water extraction for 1 h at 99°C left less than 10% of *T. weissflogii* particulate glucans on the filter. For *O. tauri*, only up to 50% of glucans were not extracted under the same conditions (Figure 3a). A longer incubation time, sequential water extractions, or a hot alkaline extraction using 1 M sodium hydroxide (1 h, 99°C) do not increase the extraction efficiency for *O. tauri*. However, a subsequent additional extraction using 1 M sodium hydroxide (1 h, 99°C) decreases the amount of residual glucose to approximately 30% (Figure 3b). Sonication of water extracted samples before incubation at 99°C gives similar results; approximately 25% residual glucose for *O. tauri* and 30% residual glucose for *P. purpureum* (Figure 3c).

The extraction was slightly improved when using 1 h sonication in 1 M NaOH followed by 1 h at 99°C. This treatment resulted in residual glucose values of 3%, 20%, and 25% in *T. weissflogii*, *O. tauri*, and *P. purpureum*, respectively (Figure 3d). However, this additional alkaline extraction did not substantially increase the efficiency of the extraction protocol. Therefore, POM samples were extracted using hot water (1 h, sonication; 1 h, 99°C) in the following sections. The integrity of laminarin and starch was also tested and confirmed by EH of starch and laminarin standards that had been subjected to extraction conditions compared to nonsubjected samples.

These results demonstrate that glucans in *O. tauri* and *P. purpureum* are more resistant to aqueous extraction methods than glucans in *T. weissflogii*. This result is probably due to different glucans present in these species. While the material of the diatom *T. weissflogii* should contain laminarin (Becker et al., 2017), the green microalgae *O. tauri* and the red microalgae *P. purpureum* should contain starch and Floridean starch, respectively (Sheath et al., 1979; Sorokina et al., 2011). Laminarin is highly soluble in water, whereas α‐glucans vary in their water solubilities; branched α‐glucans like amylopectin and glycogen have a high solubility and linear amylose has a low solubility. The differences in extraction efficiency should be taken into account with regard to glucan quantifications as the true content might be higher.

### 3.5 **α**‐Glucans and laminarin can be quantified in parallel in particulate matter extracts from cultivated microalgae

To investigate the proportion of α‐ and β‐glucans in the aforementioned three microalgae, enzymatic α‐glucan and laminarin hydrolysis assays have to be used. Three non‐axenic cultures of each of the microalgae *O. tauri*, *P. purpureum*, and *T. weissflogii* were maintained at a constant temperature of 15°C, with a 12‐h/12‐h light/dark cycle, without stirring. Samples from each culture were taken over 20 days and filtered onto glass fiber filters.

**Figure 4:**
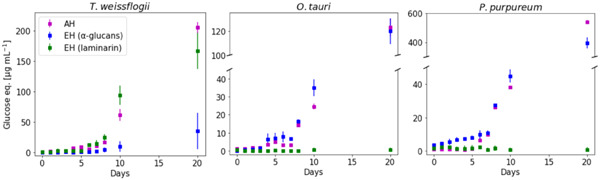
α‐Glucans can be quantified alongside laminarin in microalgal extracts using glucan‐specific enzymatic hydrolysis: glucan quantification in POM from *Thalassiosira weissflogii*, *Ostreococcus tauri*, and *Porphyridium purpureum* laboratory cultures expressed as glucose equivalents. Laminarin and α‐glucans were quantified in microalgal POM extracts using enzymatic hydrolysis and subsequent PAHBAH reducing sugar assay (EH, blue and green symbols). Total glucan was quantified by acid hydrolysis and subsequent PAHBAH reducing sugar assay (AH, magenta symbols). The amount of glucose equivalents in each sample was calculated using glucose calibration curves. Error bars denote standard deviation (*n* = 3, biological replicates). AH, acid hydrolysis; POM, particulate organic matter.

Glycans were extracted from these filters using sonication and hot water (as described above) and hydrolyzed by AH or EH. Figure 4 shows the glucose equivalents as determined by the reducing sugar assay over the course of 20 days of algal growth.

Laminarin was detectable in *T. weissflogii* diatom cultures after 6 days, but could not be detected in cultures of green microalgae *O. tauri* and red microalgae *P. purpureum* over the 20 days incubation period. Conversely, α‐glucans were detected in *O. tauri* and *P. purpureum* from Day 4 or 3, respectively. Overall, these findings match the polysaccharide profiles of these three microalgae (Becker et al., 2017; Sheath et al., 1979; Sorokina et al., 2011). However, a small amount of α‐glucans could also be measured in two of the three *T. weissflogii* cultures on Days 10 and 20. As the algal cultures were nonaxenic, it is possible that these α‐glucans originate from bacteria.

Overall, AH produces a similar total glucose signal as both enzymatic assays combined. Since the PAHBAH assay would be reactive to all reducing sugars released by AH this indicates that α‐glucans and laminarin (a β‐glucan) are the predominant glycans in these algal species and under the culture conditions and duration used here.

**Figure 5:**
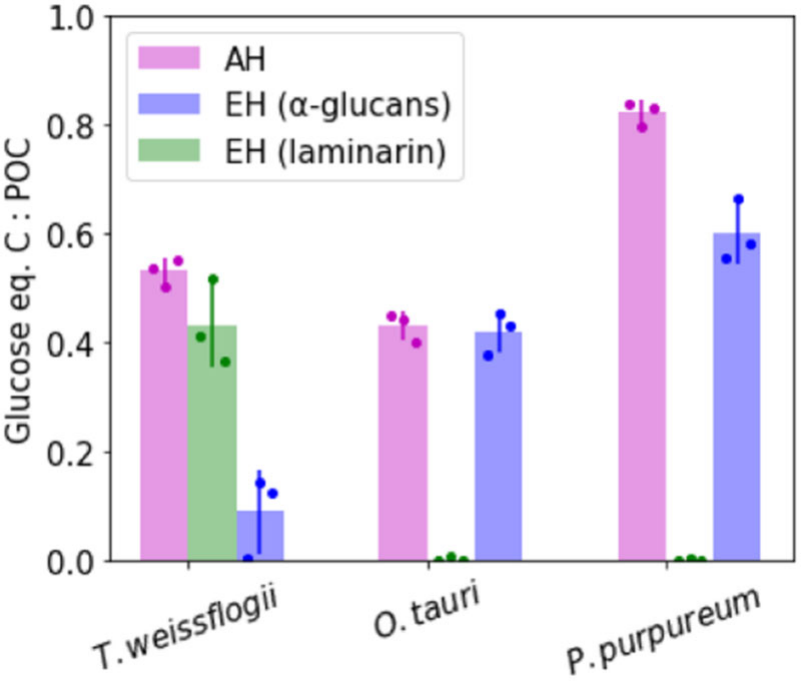
α‐Glucans account for a substantial amount of the total POC in red and green algae: α‐Glucan and laminarin quantification of microalgal POM from *Thalassiosira weissflogii*, *Ostreococcus tauri*, and *Porphyridium purpureum* laboratory cultures after 20 days of growth. Laminarin and α‐glucans were quantified in microalgal POM extracts using enzymatic hydrolysis and subsequent PAHBAH reducing sugar assay (EH, blue and green). Total glucan was quantified by acid hydrolysis and subsequent PAHBAH reducing sugar assay (AH, magenta). The amount of glucose equivalents in each sample was calculated using glucose calibration curves. α‐Glucan quantities are shown as glucose equivalent carbon relative to the total POC. Error bars denote standard deviation (*n* = 3, biological replicates). AH, acid hydrolysis; EH, enzymatic hydrolysis; POC, particulate organic carbon.

Figure 5 shows the glucan quantification on Day 20 in relation to the total POC. It should be noted that POC was quantified directly off glass fiber filters without prior extraction, whereas for glucan quantification the filters were extracted by sonication and hot water. As this extraction might not be complete, the true glucan (especially α‐glucan‐) content might be higher than depicted.

For *T. weissflogii*, about 40% of the total POC can be assigned to laminarin. Another additional 10% of POC is detectable in two of three cultures as reducing sugars released by AH. This 10% is hydrolyzed by enzymatic α‐glucan hydrolysis and probably originates from bacterial glycogen. About 40% of the POC of *O. tauri* corresponds to α‐glucans. In this case, the reducing sugars hydrolyzed by acid equal the enzymatically hydrolyzed α‐glucans. The POC of *P. purpureum* after 20 days of algal growth can largely be contributed to glycans as approximately 80% of POC are AH products. However, only about 60% of *P. purpureum* POC is hydrolyzed by the α‐glucan assay. This discrepancy might be due to a higher content of monomeric glucose or other polysaccharides only hydrolyzed by AH and none of the enzymatic assays.

In summary, around 40%–80% of POC in the tested microalgal cultures can be ascribed to glucose coming from glucans. The glucans in *T. weissflogii* are mostly laminarin (β‐glucans), and the glucans in *O. tauri* and *P. purpureum* are mostly starch‐like α‐glucans.

### 3.6 α‐Glucans and laminarin can be quantified in parallel in marine environmental particulate organic matter

Marine environmental POM samples were taken from the western North Atlantic Ocean (40°53.7′ N, 60°11.9′ W) in May 2019 and in the North Sea (54°11.3′ N, 7°54.0′ E) near Helgoland (Germany) in April 2020. In both cases, POM in surface water was filtered onto 0.7 μm glass fiber filters. The POC content was quantified directly from nonextracted filter‐circle‐cutouts, while the glucan content was determined using AH or EH of hot water extracts of quarter filters. The glucose content in the samples was found to be too low for quantification by the photometric reducing sugar assay. Therefore, HPAEC‐PAD was used to determine the glucose content in hydrolyzed and non‐hydrolyzed extract samples. With HPAEC‐PAD, as little as 0.3 and 0.2 μg/ml glucose could be detected following AH or EH, respectively, of a starch standard sample. In comparison, the quantification limit for the reducing sugar PAHBAH assay was 11 μg/ml.

**Figure A6:**
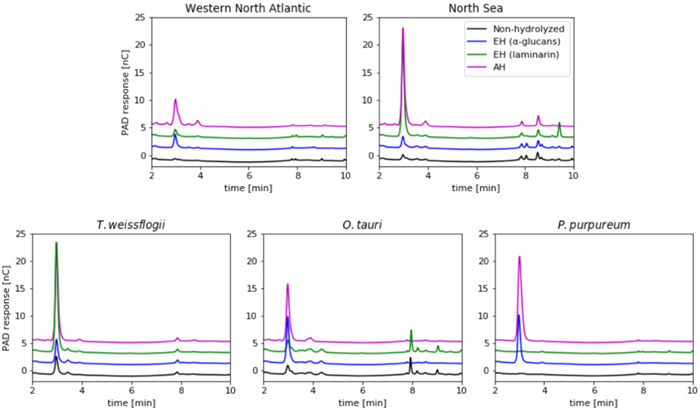
Enzymatic hydrolysis assays for aqueous extracts produce glucose: HPAEC‐PAD chromatograms (PA100 column) of extracts from (top) environmental POM samples, (bottom) algal cultures. Chromatograms of hydrolyzed samples were moved by 2, 4, and 6 to improve clarity. AH, acid hydrolysis; EH, enzymatic hydrolysis; POM, particulate organic matter.

HPAEC‐PAD analysis of enzymatically hydrolyzed α‐glucans and laminarin shows that the only product is glucose (Figure A4). Therefore glucose can be used as a standard to quantify and compare α‐glucans and laminarin. Similar chromatograms of hydrolyzed and nonhydrolyzed extracts from algal cultures and environmental samples are depicted in Figure A6.

**Figure 6:**
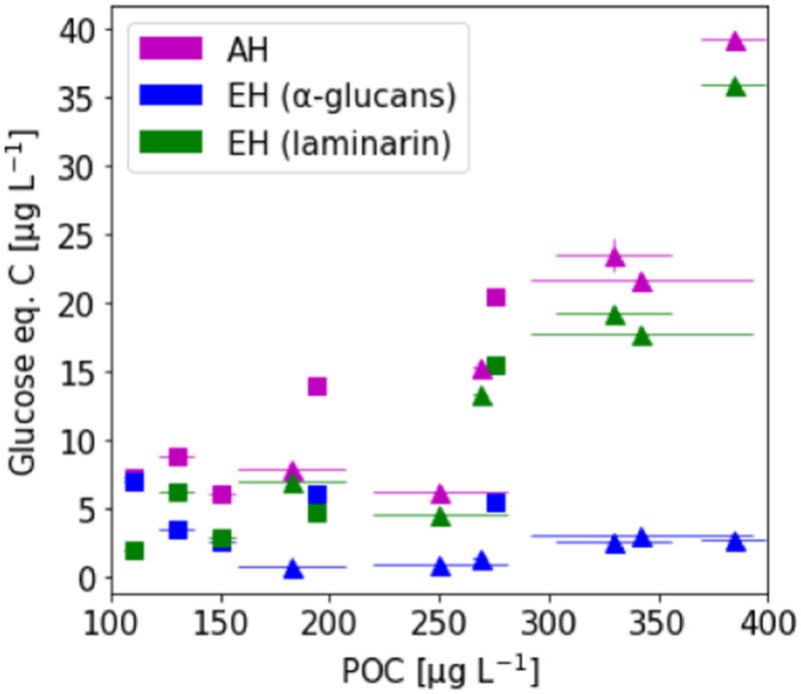
α‐Glucans can be quantified alongside laminarin in marine POM samples using glucan‐specific enzymatic hydrolysis: POM samples on 0.7 μm glass fiber filters were extracted and hydrolyzed using acid hydrolysis (AH) (magenta symbols) or enzymatic hydrolysis (EH) (blue and green symbols). Square symbols represent samples from the western North Atlantic Ocean (May 2019). Triangle symbols represent samples from the North Sea (April 2020). Error bars denote standard deviation (*n* = 3, technical replicates). POM, particulate organic matter.

Figure 6 shows the results of glucan quantification of environmental POM samples in relation to the total POC. While laminarin content in POM samples from the western North Atlantic Ocean varies between 2 and 16 μg/L carbon, it is higher in most of the North Sea samples (4–36 μg/L laminarin carbon). This is in agreement with the abundance of microalgae at the time of sampling: North Sea samples were taken during the 2020 phytoplankton spring bloom, while the samples from the Atlantic Ocean were harvested in oligotrophic waters. Overall, less α‐glucan than laminarin was detected in the environmental samples (3–7 μg/L, West Atlantic and 1–4 μg/L, North Sea). The combined glucose value for laminarin and α‐glucan, determined with EH, is similar to the total glucose value determined with AH. This observation is consistent with the results shown above (Figures 4 and 5). This result further indicates that most glucose‐containing polysaccharides are detected by one of the two enzymatic assays. Laminarin content in POM appears to correlate significantly with increasing total POC ($R^{2}$ = 77.5%, p<0.001)—in accordance with previous studies (Becker et al., 2020)—, whereas α‐glucan content is lower but apparently noncorrelating with total POC ($R^{2}$ = 9.4%, p>0.360). It should be noted that these α‐glucans may originate not only from starch‐producing red and green algae but also from glycogen‐producing bacteria.

## 4 DISCUSSION

A method for the detection and quantification of starch‐like α‐glucans in the ocean has been developed and applied to microalgal cultures and environmental marine POM. Using this method enabled the parallel quantification of α‐glucan and laminarin (Becker et al., 2017) in marine POM for the first time. While there are other methods to analyze the structure of glycans based on derivatization (Price, 2007), partial chemical lysis combined with mass spectrometry (Amicucci et al., 2020), or spectroscopy (Battistel et al., 2014), their glycan‐specific quantification in environmental samples at low concentrations is difficult (Lang et al., 2014).

This method is highly specific and robust. However, the assay cannot distinguish between the different types of α‐1,4/1,6‐glucans and is therefore not suitable for determining the origin of the α‐glucans detected. To further investigate this question, the degree of α‐1,6‐branching could also be determined by adding an endo‐acting α‐1,6‐isoamylase to the assay (Figure A2). Knowing the average degree of α‐1,6‐branching in POM glucans does not unequivocally identify the type of α‐glucans detected, but it might allow for an estimate if the main α‐glucan is the highly branched glycogen. Another approach to identify α‐glucan types could be the application of separation techniques based on different solubilities. This might allow for determining the origin of α‐glucans as starch from red (Yu et al., 2002) and green algae (Busi et al., 2014) or as bacterial storage glycogen (Ball & Morell, 2003).

Hot water extraction from POM is probably not complete (El Halal & Kringel, 2019) for all types of α‐glucans, and thus the true α‐glucan content of the environmental POM samples may be higher than the one detected. However, the extraction approach used in our method allows the parallel detection of other polysaccharides, such as laminarin (Becker et al., 2020), by enzymatic assays.

This study has highlighted problems in quantifying glucans after chemical lysis, as AH consistently produced fewer glucose products from polysaccharide standards than enzymatic hydrolysis. It is known that different hydrolytic conditions result in different hydrolysis yields for distinct polymers within a sample (Zhu et al., 2020). The hydrolysis efficiency depends on the hydrolysis method as well as the carbohydrate types and even after harsh hydrolysis conditions some polysaccharides do not hydrolyze into monosaccharides (Panagiotopoulos & Sempéré, 2005; Zhu et al., 2020). Despite this problem, the information about the monosaccharide composition provided by non‐specific glycan acid hydrolysis is still very valuable for complex samples (Engel & Händel, 2011).

α‐Glucan concentrations in marine surface water POM in our samples were found to be lower than laminarin but at constant levels, independent of increased POC concentrations during the 2020 North Sea seasonal algal bloom. To determine the relevance of α‐glucans in the marine carbon cycle more glycan‐specific data need to be collected. As bacterial storage glycogen, α‐glucans could be an important indicator of bacterial carbon turnover (Kappelmann et al., 2019). In addition, higher α‐glucan concentrations are expected near coasts and estuaries due to the input of terrestrial plants and locations with distinct green and red algal growth. However, it is likely that in marine glycan samples, α‐glucan concentrations are usually lower than laminarin concentrations (Becker et al., 2020). This low concentration of specific types of glycans in the marine carbon pool is a major obstacle to the analysis of marine glycans by EH and requires the use of appropriate detection methods with low detection limits, such as HPAEC‐PAD (Lo‐Guidice & Lhermitte, 1996), mass spectrometry (Hofmann & Pagel, 2017), or fluorometric methods (Mutuyimana et al., 2018; Tran et al., 2021) for glucose quantification.

The lack of knowledge about the specific carbohydrate structures that are present on the sea surface and the ones that are exported to the deep sea remains a serious hindrance to our understanding of the role of glycans in the marine carbon cycle. We need to analyze marine samples with methods that allow high structural resolution. The method described in this study allows the specific identification of α‐glucans. We envisage that this method will be one of many glycan quantification methods based on EH for environmental samples.

## AUTHOR CONTRIBUTIONS

**Nicola Steinke**: Conceptualization (lead); data curation (lead); formal analysis (lead); investigation (lead); methodology (lead); project administration (lead); validation (lead); visualization (lead); writing – original draft (lead); writing – review and editing (lead). **Silvia Vidal‐Melgosa**: Investigation (supporting), writing – review and editing (supporting). **Mikkel Schultz‐Johansen**: Investigation (supporting), writing – review and editing (supporting). **Jan‐Hendrik Hehemann**: Funding acquisition (lead), investigation (supporting), resources (lead), writing – review and editing (supporting).

## CONFLICTS OF INTEREST

None declared.

## ETHICS STATEMENT

None required.

## Figures and Tables

**Figure A5:**
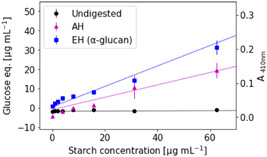
Figure 1, zoomed‐in on lower starch concentrations: enzymatic hydrolysis (EH) products of starch, acid hydrolysis (AH) products of starch, nondigested starch, and glucose as calibration standard were measured in α‐glucan assay buffer (100 mM sodium acetate buffer, pH 4.5) using the PAHBAH reducing sugar assay. $A_{410\text{nm}}$ signals above 1.9 were measured in diluted samples. Error bars denote standard deviation (*n* = 3, technical replicates). Solid lines represent regression lines.

## Data Availability

All data are provided in full in this paper.

## APPENDIX A

**Table A1:** Polysaccharide standards used for the results depicted in Figures 1, 2, A3, A4.

Polysaccharide	Source	Monosaccharide(s)	Glycosidic linkages
Starch*	Corn	Glucose	α‐1,4, α‐1,6
Amylopectin*	Corn	Glucose	α‐1,4, α‐1,6
Amylose*	Potato	Glucose	α‐1,4
Glycogen*	Oyster	Glucose	α‐1,4, α‐1,6
Pullulan**	*Pullularia pullulans*	Glucose	α‐1,4, α‐1,6
Laminarin*	*Laminaria digitata*	Glucose	β‐1,3, β‐1,6
Laminarin***	*Eisenia bicyclis*	Glucose	β‐1,3, β‐1,6
Lichenan**	Icelandic moss	Glucose	β‐1,3, β‐1,4
β‐Mannan**	Ivory nut	Mannose	β‐1,4
β‐Glucan**	Barley	Glucose	β‐1,3, β‐1,4
Polygalacturonic acid**	Citrus pectin	Galacturonic acid	α‐1,4
Xyloglucan**	Tamarind	Xylose, Glucose, Galactose, Arabinose	β‐1,4, α‐1,6, β‐1,6
Carboxymethyl cellulose**	Cellulose	Glucose	β‐1,4
α‐Mannan*	Yeast	Mannose	α‐1,6, α‐1,2, α‐1,3

* Sigma‐Aldrich.

** Megazyme.

*** Carbosynth.
